# Extending Porous Silicone Capacitive Pressure Sensor Applications into Athletic and Physiological Monitoring

**DOI:** 10.3390/s21041119

**Published:** 2021-02-05

**Authors:** Yun Xia, Hao Gu, Lei Xu, Xiao Dong Chen, Tim V. Kirk

**Affiliations:** Suzhou Industrial Park Campus, School of Chemical and Environmental Engineering, College of Chemistry, Chemical Engineering and Material Science, Soochow University, Suzhou 215123, China; 20154009042@stu.suda.edu.cn (Y.X.); 20194209288@stu.suda.edu.cn (H.G.); 20184209291@stu.suda.edu.cn (L.X.); xdchen@mail.suda.edu.cn (X.D.C.)

**Keywords:** capacitive pressure sensor, wearable sensor, porous dielectric, sleep monitoring, physiological monitoring, athletic monitoring

## Abstract

Porous polymer dielectric materials have been developed to increase the sensitivity of capacitive pressure sensors, so that they might expand capacitive sensor use, and promote the realization of the advantages of this class of sensor in further fields. However, their use has not been demonstrated in physiological monitoring applications such as respiration monitoring and body position detection during sleep; an area in need of unmet medical attention for conditions such as sleep apnea. Here, we develop and characterize a sensor comprised of a poly dimethylsiloxane (PDMS) sponge dielectric layer, and PDMS/carbon black (CB) blend electrode layers, with suitable compliance and sensitivity for integration in mattresses, pillows, and athletic shoe insoles. With relatively high pressure sensitivity (~0.1 kPa^−1^) and mechanical robustness, this sensor was able to fulfill a wide variety of roles, including athletic monitoring in an impact mechanics scenario, by recording heel pressure during running and walking, and physiological monitoring, by detecting head position and respiration of a subject lying on a pad and pillow. The sensor detected considerably greater relative signal changes than those reported in recent capacitive sensor studies for heel pressure, and for a comparably minimal, resistive sensor during respiration, in line with its enhanced sensitivity.

## 1. Introduction

Strain and pressure sensors derived from soft materials have been pursued for a number of years, as their flexibility [1], compliance [1,2], and morphological variety [3,4,5] have made them attractive for integration into a number of applications, particularly for biosensing roles with wearable devices [6,7,8,9,10,11,12], and human motion monitoring [13,14,15,16,17].

Soft material sensors are typically divided into two classes: piezoresistive and capacitive [18]. Resistive sensors were developed for many applications due to their typically high sensitivity to pressure and strain [19,20,21,22], and significant durability has been demonstrated in some high sensitivity systems in recent times [23]. Capacitive sensors, with their restriction of conductive elements to electrodes for interrogation of dielectric layers [3,24], have potential for mechanical robustness, particularly with large deformation. This class has demonstrated fast responsiveness [4,25,26], good linearity [4,5,25,26], low hysteresis [4,5,25,26], durability [4,27,28], and insensitivity to changes in temperature and humidity [28,29], but may lack sensitivity [20,22]. For wearable applications, where sensors are expected to undergo many high strain or compression cycles, and even impact, this robustness may be particularly important. A brief survey of various capacitive pressure sensors is given in Section 3.

Recently, porous polymer capacitive sensors have been developed to increase pressure sensitivity and expand applications for this class of sensor [12,29,30]. Generally based on porous silicone dielectric elements, they have increased sensitivity from the typical order of ~1 to ~100 MPa^−1^, while using relatively inexpensive materials. The material components, performances, and applications of these sensors are surveyed in Section 3.

Introduced here, is a polymer capacitive sensor with a flexible dielectric layer based on a poly dimethylsiloxane (PDMS) sponge, and a carbon black (CB)/PDMS blend for its flexible electrodes. The sponge was produced via selective leaching of citric acid monohydrate (CAM) particles, and was then coated with CB/PDMS blend electrode layers, and subsequent PDMS packaging layers via multilayer casting and assembly. Core parameters of pressure sensing were evaluated, and durability over 10,000 cycles of tensile and compression cycling was established. Multiplexing was demonstrated via integration into a pressure mat array, while basic wearable use was established through four point pressure monitoring, with a band during flexing of a human elbow. Extension into athletic monitoring in an impact mechanics scenario was shown via monitoring of heel pressure, during running and walking, with an insole integrated sensor, and a new application in physiological monitoring was achieved by monitoring head position and respiration with three sensors integrated into a pillow and a mattress, respectively.

Insole sensing of plantar pressure, and subsequent gait analysis, has been dominated by resistive sensors, commonly referred to in that field as force sensitive resistors (FSRs) [2]. Polymer capacitive sensors are considered to have advantages in terms of low power consumption, detection resolution, and simplicity of large area fabrication [31]. Conversely, concerns about use of FSRs for insole applications have been raised with regard to signal and calibration stability [32], suitability for integration, and mechanical properties [2]. Given the shock absorbing nature of athletic shoe insoles, the heat and humidity produced in a shoe during exercise, and the greater sensitivity of resistive sensors, this represents an opportunity for compliant porous silicone based capacitive materials to compete with resistive elements. In 2017, Tolvanen et al. [33] introduced a highly compliant polyurethane (PU) foam based capacitive sensor, and attached it to the heel of an insole. Here, we demonstrate that porous silicone sensors can fulfil this role as well.

Typically, sleep behavior is assessed using a complex, multi-sensor method known as polysomnography (PSG), which is performed at specialist facilities [34]. Simplified systems that do not interfere with normal sleep via cumbersome instrument attachment, and can be used in the home, represent unmet medical need for conditions such as sleep apnea. Monitoring has been pursued via methods such as pressure sensor array mattresses [34,35,36], with as many as 320 sensors [34], and pressure sensor integrated pillows [37,38], with as few as three sensors [38]. Key behaviors to monitor during sleep include respiration and body position, with respiration potentially requiring high sensitivity to monitor with only a few sensors embedded in a soft material. Indeed, reports of the performance of commercial FSRs have only shown a few percent signal change during respiration [39]. Relatively few studies concerning materials aspects of sleep monitoring have been published, but recently, Tian et al. [38] placed three textile based, piezoresistive pressure sensors beneath a pillow, and detected respiration and an indicator of sleep position with this simplified system. Here, we have integrated three of the porous silicone based capacitive sensors with a pillow for detection of head position, and have attached three across a pad to monitor respiration; a new application for this class of dielectric materials, made possible by their relatively high sensitivity.

## 2. Experimental Details

### 2.1. Materials and Fabrication Apparatus

Cabot XC-72R Carbon Black (CB) powder was purchased from Cabot Corporation (Boston, MA, USA); Sylgard-184 PDMS (silicone elastomer base and silicone elastomer curing agent) from Dow Corning Company (Midland, MI, USA); ethanol and isopropanol from Sinopharm Company (Shanghai, China); citric acid monohydrate (CAM) was purchased from Titan Corporation (Shanghai, China); polyethylene terephthalate (PET) sheets of 0.05 and 0.2 mm thickness were purchased from Shihua company (Suzhou, China). The automatic film applicator (AFA-II) was purchased from Shanghai Moderner Company (Shanghai, China), and a height adjustable stopper (HAS) (1806B) from BEVS Company (Shanghai, China).

### 2.2. Fabrication of Sensor

**Figure A1:**
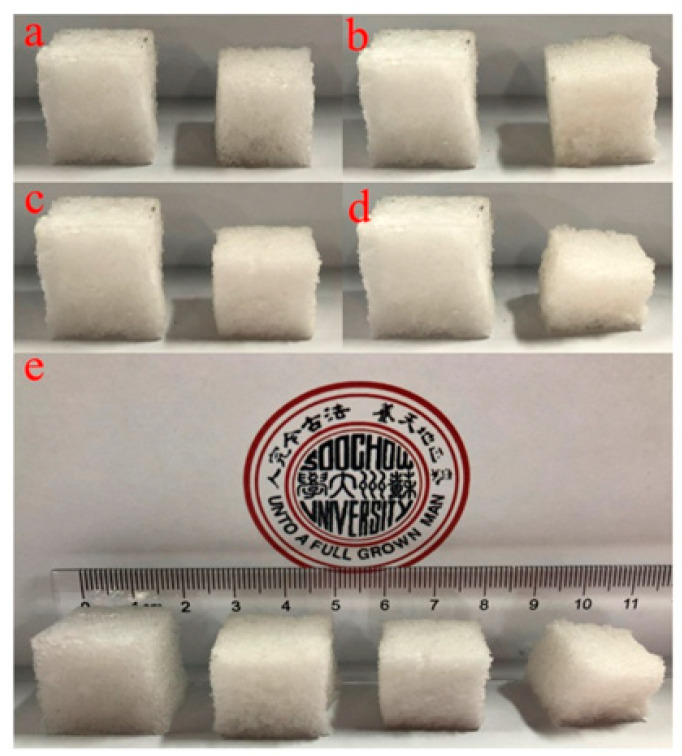
The images of the PDMS sponge with different mass fraction of CAM. (**a**) m_PDMS_:m_CAM_ = 1:3, (**b**) m_PDMS_:m_CAM_ = 1:5, (**c**) m_PDMS_:m_CAM_ = 1:7, (**d**) m_PDMS_:m_CAM_ = 1:9, (**e**) the images of four PDMS sponges.

For the dielectric sponge, PDMS base and curing agent were mixed at a mass ratio of 20:1, as suggested in the literature [40], and stirred for 5 min, then citric acid monohydrate (CAM) and the PDMS were combined at a mass ratio of 7:1 (see in Appendix A Table A1 and Figure A1), and poured into a mold with ~5 mm depth, and placed in a blast oven at 60 °C to cure. For the conductive silicone electrode layers, PDMS base and curing agent were mixed at a mass ratio of 20:1, stirred for 5 min, then combined with carbon black (CB) at CB mass fraction of 25%, and stirred well for 3 min. This mixture was then coated on a PET film. The automatic film applicator and height adjustable stopper were used to cast a PDMS/CB conductive film ~200 µm thick. This was placed in an oven at 70 °C to cure. Finally, the flexible conductive film was peeled from the PET sheet. PDMS only layers were produced with a base:curing agent ratio of 20:1.

**Figure 1:**
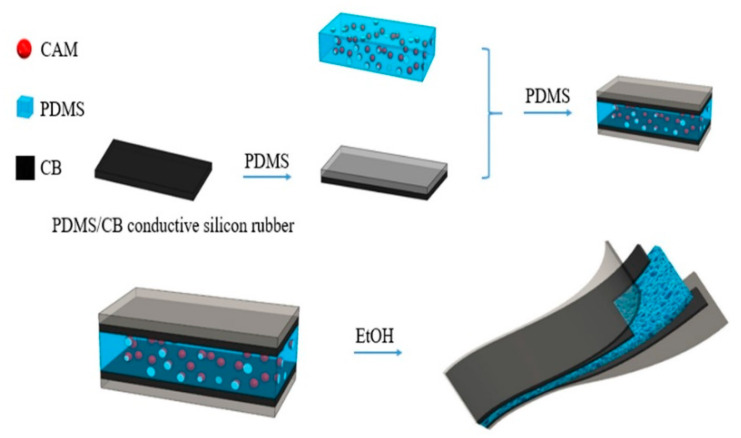
Schematic diagram of the sensor layers and materials, and their assembly.

Figure 1 shows the assembling process for the sensor layers. Briefly, the first PDMS/CB layer was coated with an ~100 µm thick PDMS layer using the film applicator, and was cured; these layers were flipped vertically and the other side of the PDMS/CB layer was coated as mentioned before. Before curing, the PDMS/CAM dieletric layer was placed on top of the uncured PDMS layer, and that layer was cured; the process was repeated for the second PDMS/CB layer, to achieve a bonded and cured stack. Finally, the sensor was placed in ethanol for 24 h, to leach out the CAM, and was then washed with water and dried in an oven.

### 2.3. Characterization

Morphology was examined using Scanning Electron Microscopy (SEM), with an SU1510 microscope from Hitachi Company (Tokyo, Japan). Large deformation durability was evaluated by reciprocating cycle straining, using a lab-made tensile straining system (0.2 Hz cycle frequency, 40% elongation), and by compression cycling (0.23 Hz cycle frequency, 40% compression) carried out on a texture analyzer (CT3 50 K, Brookfield, Middleboro, MA, USA) at a test speed of 0.5 mm·s^−1^. Pressure sensitivity testing was also carried out on the texture analyzer, over a range of 14 kPa. Single capacitance signals were measured by a capacitance meter (TH2638, Tonghui, Changzhou, China) at ~70 ms sample period, with a 10 kHz frequency signal. A multiplex and wearable device capacitance signals were measured using a lab-made capacitance meter, based upon the PCAP01 (Acam-Messelectronic Gmbh, Stutensee, Germany) integrated circuit, at 2.5 Hz (400 ms sample period).

### 2.4. Device Integration

The four sensor pressure mat shown in Figure A4 was assembled as described above, but the sensors were placed upon a PET sheet and covered with uncured PDMS liquid at a thickness such that the top surface was 1 mm above the sensors; the PDMS was cured, and the 1 mm thick PDMS layer formed the mat.

The elbow band shown in Figure A5 was the same four channel pressure mat from above, and was attached to the table via medical tape.

The insole heel pressure sensor, shown in Figure A6, was assembled as follows: a hole was cut through the insole and the sensor was inserted, then the top and bottom surfaces were covered with glue and cotton fabric to seal the sensor in place. For this application C_0_, the reference capacitance, was taken as the lowest capacitance measured during each exercise experiment.

For integration with the pillow, shown in Figure A7, a 490 mm × 280 mm × 5 mm polyurethane (PU) foam mat was modified as follows: three equally spaced holes, approximately sensor deep, were made across the lateral center line of the mat; these were filled with sensors, and covered with glue and cotton fabric. This sensor mat was then secured to the pillow underside with double-sided tape.

The three sensor PU mat was used to simulate a mattress, as shown in Figure A9.

For all wearable device or physiological monitoring experiments a single subject—the author—used the devices.

## 3. Result and Discussion

A performance survey of published sensor materials, that lack a porous silicone dielectric layer, is presented in Table 1. Notable, are the low sensitivities of most capacitive pressure sensors—generally on the order of 1 MPa^−1^. The outstanding exception, from Wan et al. [41], utilized a GO foam film as its dielectric layer, achieving a sensitivity over the 10 kPa range of 150 MPa^−1^, similar to the 49 to 100 MPa^−1^ result reported here. When characterized for strain sensitivity, GF values from ~0.4 to 1 were reported. The 0.51 GF of the report’s sensor lies within a common range for materials primarily intended for pressure measurement. Typically, pressure sensors have not been extensively characterized for durability, with ~1000 cycles of deformation common, compared with the 10,000 to 100,000 cycles sometimes reported for strain sensors [28]. The 10,000 strain and pressure cycles evaluated here are relatively high for capacitive pressure sensors.

Immediately apparent from Table 2, is the much higher sensitivity displayed by porous silicone dielectric based sensors—values in the 100 MPa range are common around 10 kPa applied pressure, with much higher sensitivities again in the lower ranges, such as <2 kPa. Several of these have been characterized for durability over 10,000 cycles, though typically this was limited to indentation cycling, rather than the more rigorous large strain cycling tests.

**Figure 2:**
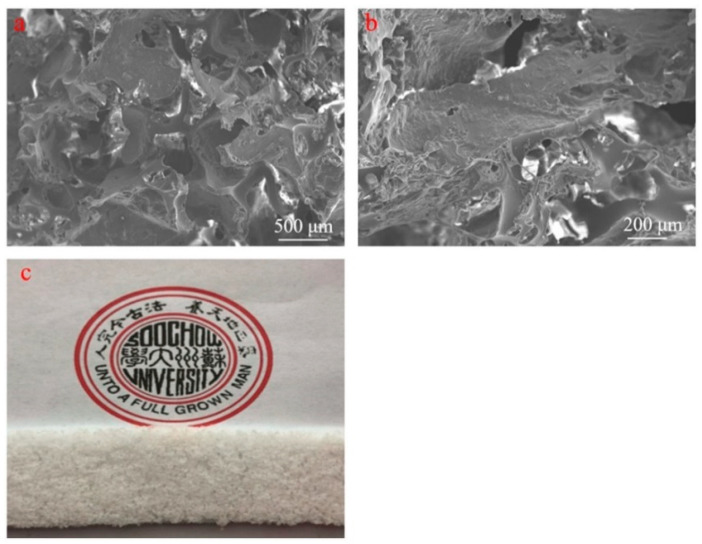
(**a**) SEM image showing the surface of the PDMS sponge (scale bar: 500 μm), (**b**) higher magnification SEM image of the PDMS sponge (scale bar: 200 μm), (**c**) photographic image of ~5 mm thick PDMS sponge.

The porous PDMS sponge shown in Figure 2a has pore sizes in the order of 500 μm. Some degree of connection between pores seems apparent from Figure 2b, indicating an at least partially open-cell configuration. The macroscopic view shown in Figure 2c shows the mechanically stable, larger scale structure of the sponge.

**Figure 3:**
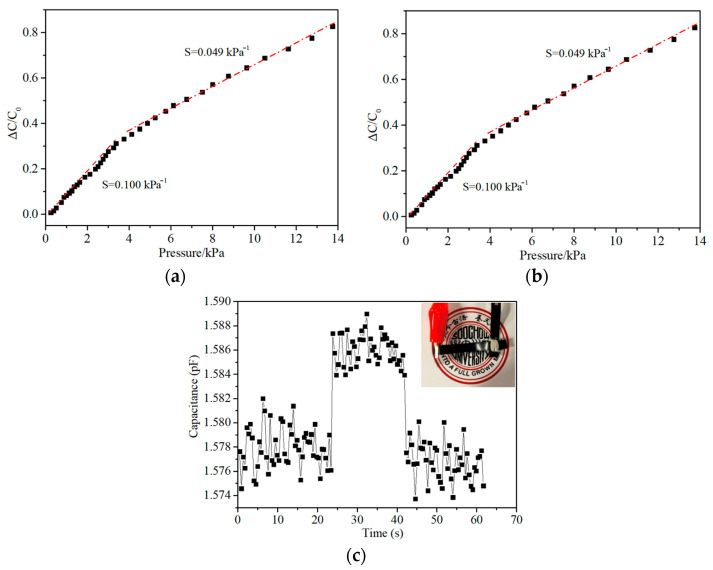
Capacitive sensing characteristics of the pressure sensor. (**a**) Relative capacitance change with respect to progressively increasing pressure. (**b**) Relative capacitance change of the pressure sensor from consecutive linear loading–unloading cycles. (**c**) Capacitive response of the pressure sensor to the placing and removal of 2 grains of rice (total mass 44 mg).

The capacitive sensing characteristics of the material are displayed in Figure 3, with sensitivity defined as S = δ(ΔC/C_0_)/δp, where p represents the applied pressure, and C and C_0_ indicate the capacitances with and without the applied pressure [4]. The relative capacitance behavior in Figure 3a displays a sensitivity of 0.100 kPa^−1^ below ~4 kPa, and 0.049 kPa^−1^ above that pressure. Reductions in sensitivity with increasing pressure are commonly observed with most sensors, and particularly so with porous silicone materials. The sensitivity displayed here, above 4 kPa, is quite typical for these materials, as shown in Table 2. However, the sensor does not display the very high sensitivity displayed by some of these materials below 1 kPa, such as the 0.63 kPa^−1^ recorded by Kang et al. [30] The more consistent but lower sensitivity, seen here, is similar to that demonstrated by Amit et al. [20]. Mild hysteresis is displayed in Figure 3b, which is typical of many capacitive pressure sensors, from porous silicone [30] to textile fiber based sensors [5]. The sensor’s ability to clearly detect the addition of a small load (44 mg), from two grains of rice, is shown in Figure 3c. Variance data via multiple samples were not collected over the sensitivity curve range, but signal stability at high compression (40%) was assessed over 10,000 cycles in durability tests.

**Figure 4:**
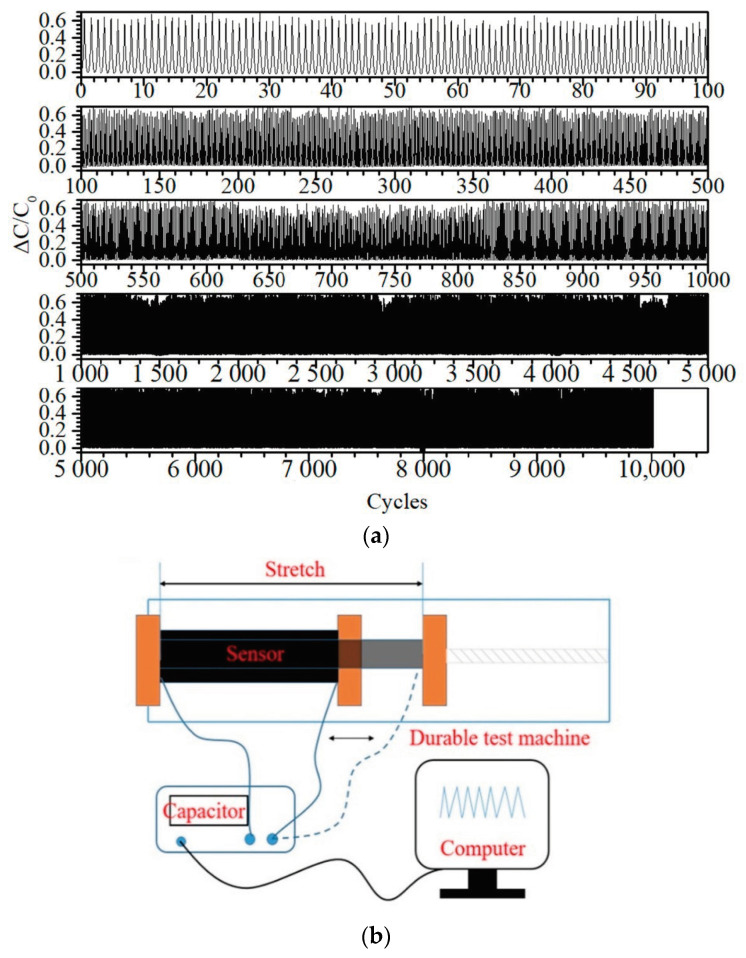
(**a**) Signal traces from tensile strain cycling, recorded at a frequency of 0.2 Hz with 40% elongation, and displayed over different periods from this durability test. The cycles shown in descending order are zero to 100, 100 to 500, 500 to 1000, 1000 to 5000, and 5000 to 10,000. (**b**) Schematic of the custom tensile strain cycling test apparatus.

**Figure A2:**
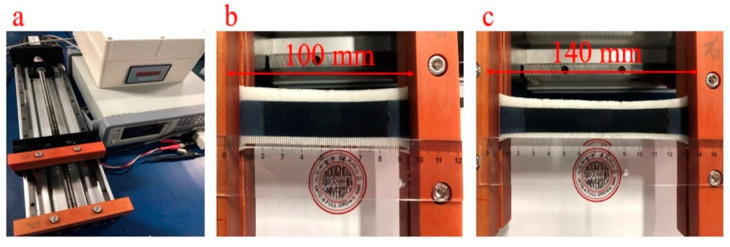
(**a**) Photo of the durable test machine, (**b**) photo of the sensor before stretch, (**c**) photo of the sensor at 40% elongation.

**Figure A4:**
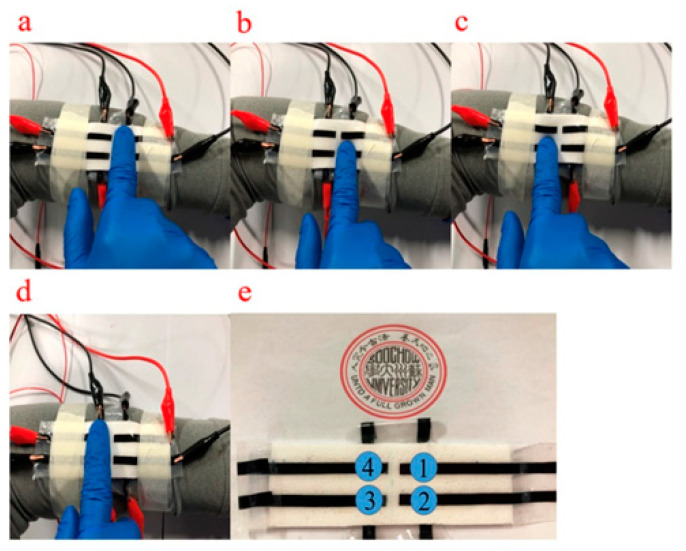
(**a**–**d**) Photos of the test when we touch the sensor. (**e**) Photo of the sensor we made.

**Figure A5:**
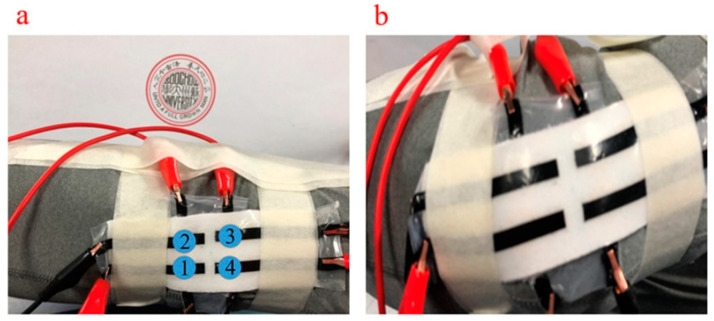
(**a**) Photo of the sensor placed on our elbow. (**b**) Photo of the sensor placed on our elbow under stretching.

Traces of relative capacitive response to strain cycling are shown in Figure 4a. Over these 40% elongation cycles the sensor’s response remains near constant at ~0.2, with little drift observable over the ranges displayed. From the comparisons in Table 1 and Table 2, it is clear that this material has demonstrated equal reliability and stability to any of the materials capable of this order of pressure sensitivity. Further details of the custom tensile testing apparatus and procedure are given in Figure A2a–c, in Appendix A, respectively.

Sensor response time, *t_r_*, has been defined as the period between the start of mechanical stimulation and the point at which the signal has risen three standard deviations above the base-line [26]. This can be estimated by linear interpolation between capacitance data points, and is shown in Figure A8 of Appendix A. Here, this gives ~80 ms, which is similar to the values typically reported for capacitive sensors [3,51]. This estimate is limited by the ~70 ms acquisition period of the apparatus used in this study, and may not fully characterize the material, given the high elastic extensibility of the silicone rubber, and the viscoelastic behavior from its air filled porous structure.

Table 3 gives the sensor’s GF at different temperature and humidity combinations. The experimental results show a minor possible decrease with rising temperature increase, consistent with the literature reports [52], and a minor potential increase in sensitivity humidity, again corresponding to the literature [53]. Overall, little change in performance is evident over these conditions, and further conclusions would require analysis of multiple samples and statistical tests.

**Figure 5:**
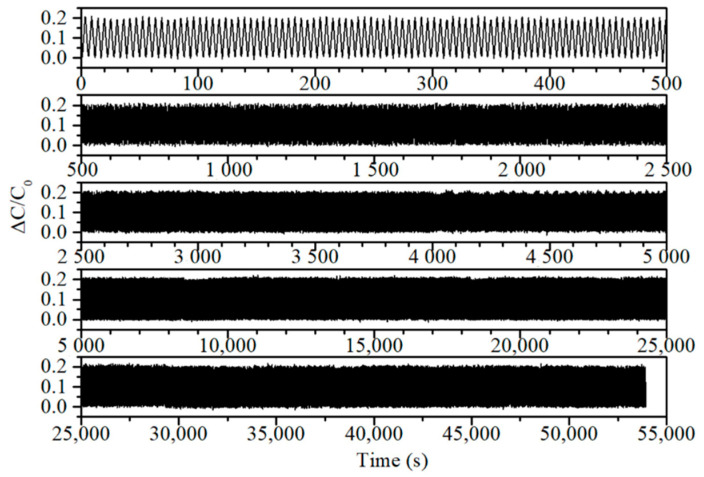
Signal traces from texture analyzer repetitive compression testing, recorded at a frequency of 0.23 Hz with 40% compression, and displayed over different periods from this durability test. The cycles shown in descending order are zero to 100, 100 to 500, 500 to 1000, 1000 to 5000, and 5000 to 10,000.

The sensor relative capacitive response to large deformation (40%) repetitive compression cycling is shown in Figure 5. Some variation between cycles is clearly evident from the peak values (~0.6), but even after 10,000 cycles typical peak values are largely unchanged. The latter observation is typical amongst the literature on porous silicone capacitive sensors [12,30,50]. However, the between peak variation has not been extensively characterized, particularly at such a large compressive deformation. As with the strain cycle testing, the material has proven to be durable over a large number of cycles.

**Figure 6:**
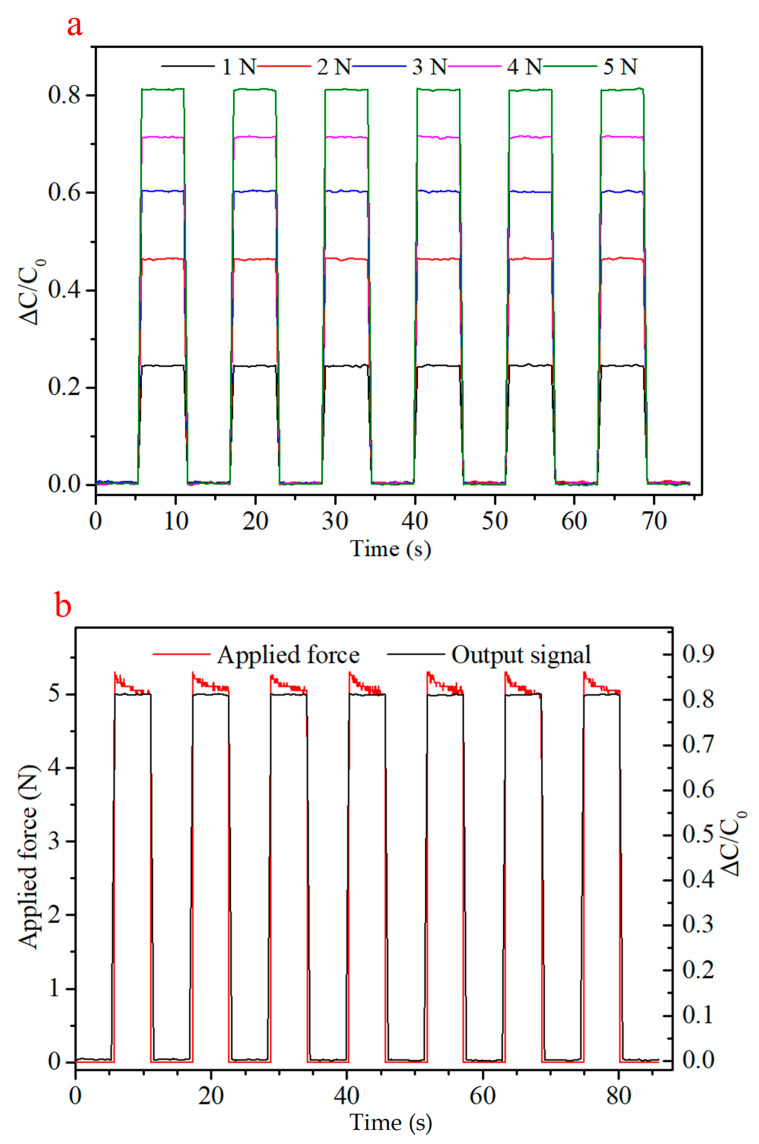
(**a**) Capacitive response of the pressure sensor to applied loads of 1, 2, 3, 4 and 5 N. (**b**) Capacitive response and mechanical relaxation for the material under repeated application and removal of a 5 N load.

Compression of porous silicone may result in varying loading or signal response. To characterize this, approximately constant compressive forces were applied to the sensor material, and signal and mechanical responses were recorded. Figure 6a shows the capacitive signal response for six applications of each force from 1 to 5 N. Signal response is consistent over time, rather than displaying an influence from changing thickness of dielectric layer. This is in keeping with the observations of Lee et al. [4]. The actual applied force did vary over time however, showing a decay-curve force relaxation over ~5 s to a steady level. This is not unexpected though, as compression of a porous material generally results in viscoelastic behavior due to the consolidation mechanism [54]. Initially, a greater force is required to expel a viscous fluid that resists compression, with this excess decaying as the material is compressed. Though this has not been previously characterized for porous silicone sensors, similar mechanical responses have been observed with a textile based capacitive sensor, again by Lee et al. [4].

**Figure 7:**
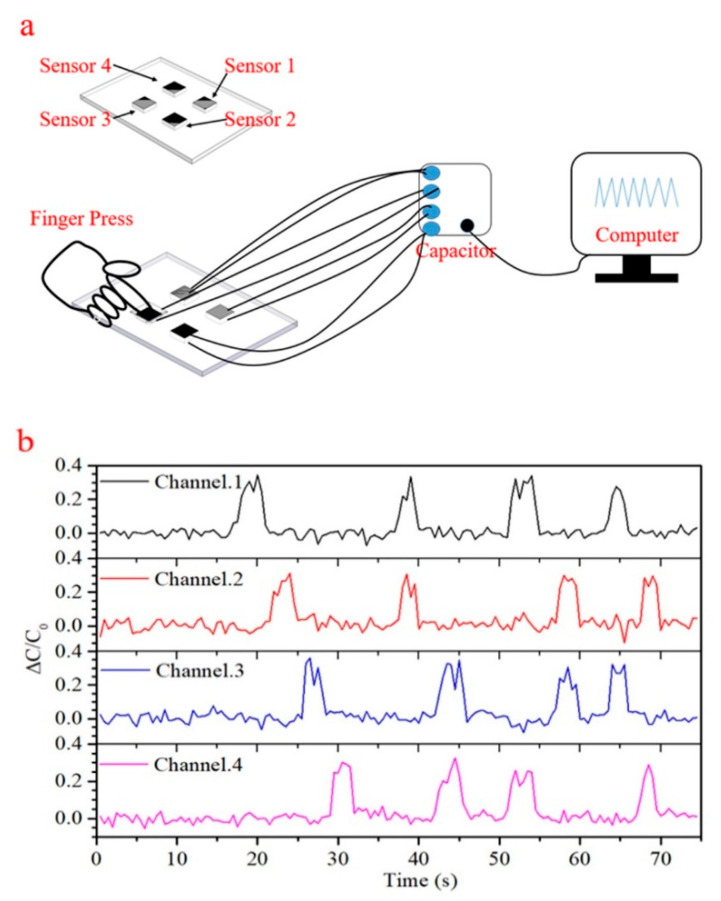
(**a**) The test circuit diagram of a four channel pressure sensing mat. (**b**) Capacitive traces demonstrating channel separation and signal multiplexing.

Simple multiplexing of the sensor was established by integration of four sensors into a pressure mat, as shown in Figure 7a. Basic functionality and channel separation is shown via the four sensor traces in Figure 7b. First, sensors one through four were pressed in sequence to demonstrate channel separation. Then clearly distinguished simultaneous multi-channel touch detection was shown by pressing pairs of sensors in the following order: one and two, three and four, one and four, two and three, one and three, and two and four.

**Figure 8:**
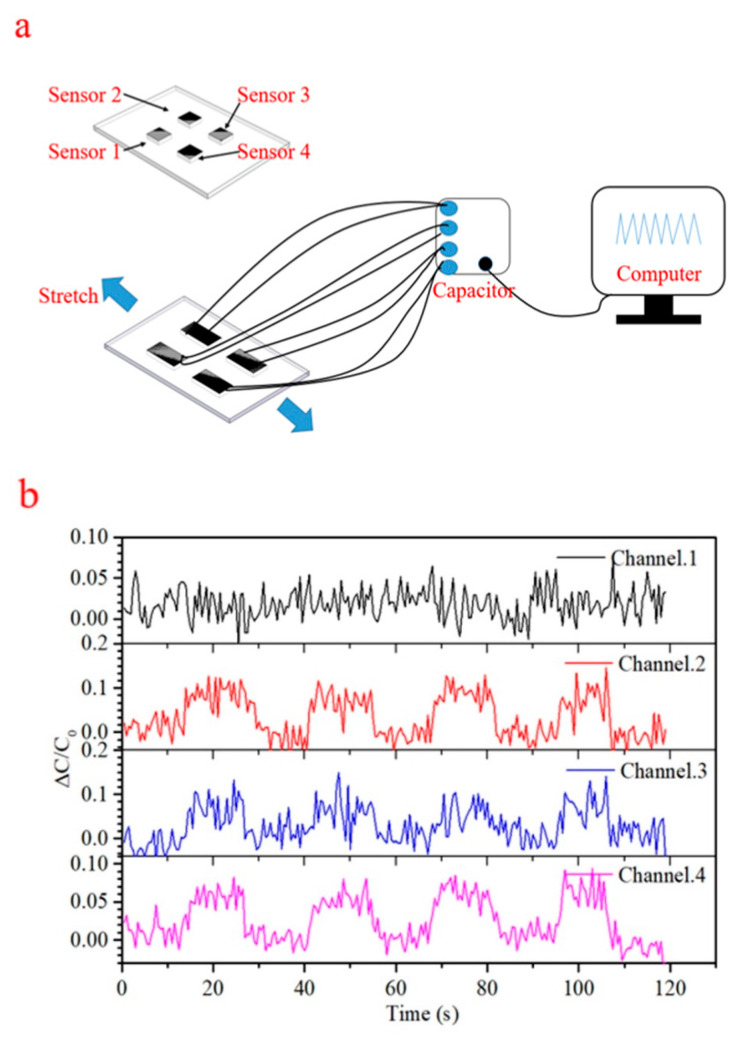
(**a**) The test circuit diagram of a four channel sensor integrated elbow band. (**b**) Capacitive traces demonstrating monitoring of elbow motion during flexion with the band.

Basic wearable sensor application was established through four point pressure monitoring with a band during flexion of a human elbow. The capacitance traces in Figure 8b illustrate pressure changes at four points on the band over four cycles of elbow flexing. As the flexion angle increases, generated pressure visibly increases, then relaxes as the elbow is straightened again. What is clear from the magnitude of the trace changes, is that pressure varies substantially across the band, perhaps indicating a biomechanical difference with position. Further development of a sensor band for elbow flexion monitoring would be required to take more detailed and defined measurements of force or pressure, but basic monitoring of human motion has been shown. For all wearable devices or physiological monitoring experiments, a single subject—the author—used the devices. As such, they represent proof-of-principle demonstrations.

**Figure 9:**
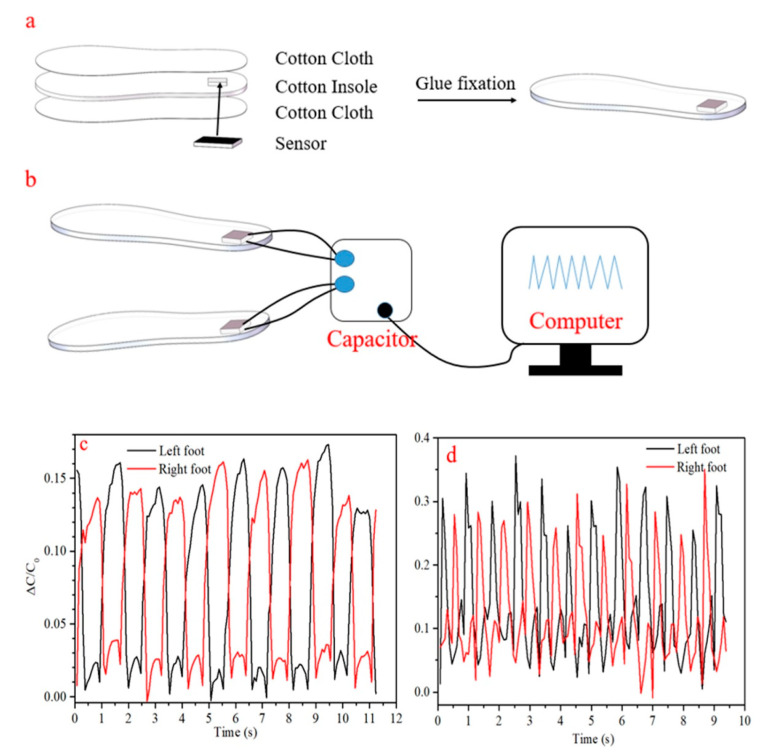
(**a**) Schematic of senor integration in the heal of an insole, (**b**) test circuit diagram for the insole sensor, (**c**) capacitive traces during walking with left and right foot insoles, (**d**) capacitive traces during running with left and right foot insoles.

Monitoring of heel pressure for both feet is given in Figure 9. Large changes in relative capacitance during walking and running are shown in Figure 9c,d, respectively, with 0.15 typical for walking, and 0.3 for running. These values are substantially larger (~3× for walking) than the few percent observed with the already relatively sensitive, minimal textile based sensor of Zhang et al. [2], befitting from the promised increased sensitivity of porous sensors. However, the highly compliant PU foam capacitive sensor reported by Tolvanen et al. [33] exhibited very high changes in capacitance (>100%), perhaps due to the high degree of compression it underwent. Additionally, capacitive sensors measure deformation of a dielectric material, rather than pressure directly, hence when a sensor is integrated with soft, deformable elements such as cotton, glue, and an athletic shoe sole, the deformation is reduced, and so is the apparent sensitivity This suggests that there may be an optimization to be made between signal change magnitude and the mechanical strength and thickness of the sensor, and its packaging or integration materials for this application.

**Figure A6:**
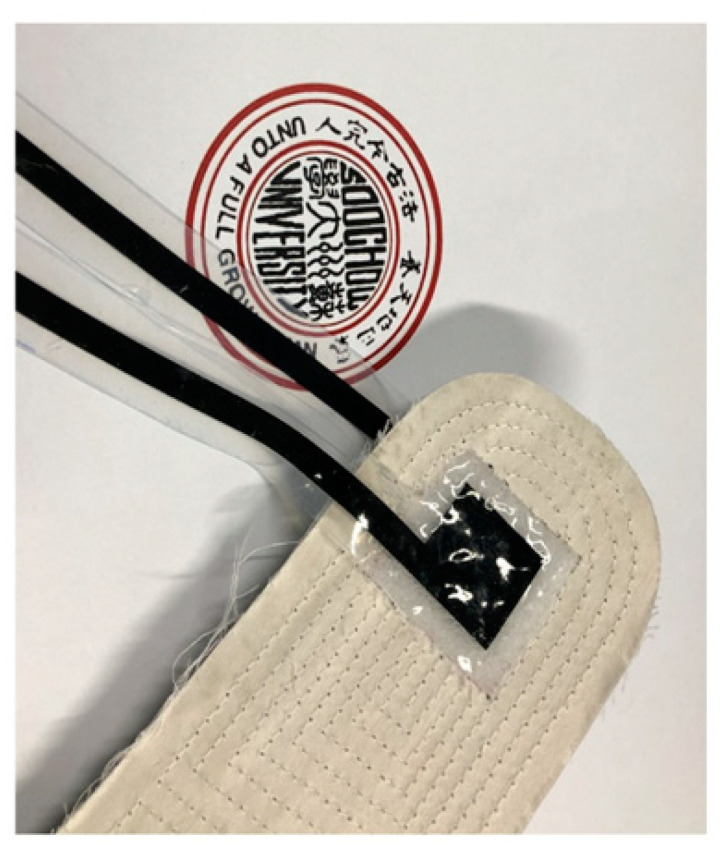
Photo of placing of the sensor on the insole.

**Figure A7:**
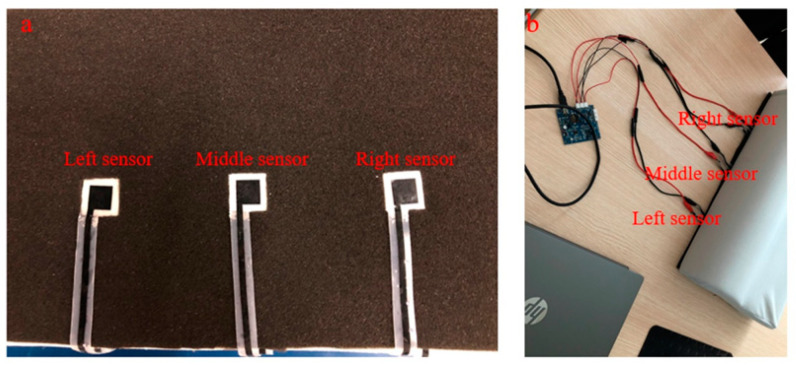
(**a**) Three flexible sensors on the pillow, (**b**) the test circuit diagram of our smart pillow.

**Figure A8:**
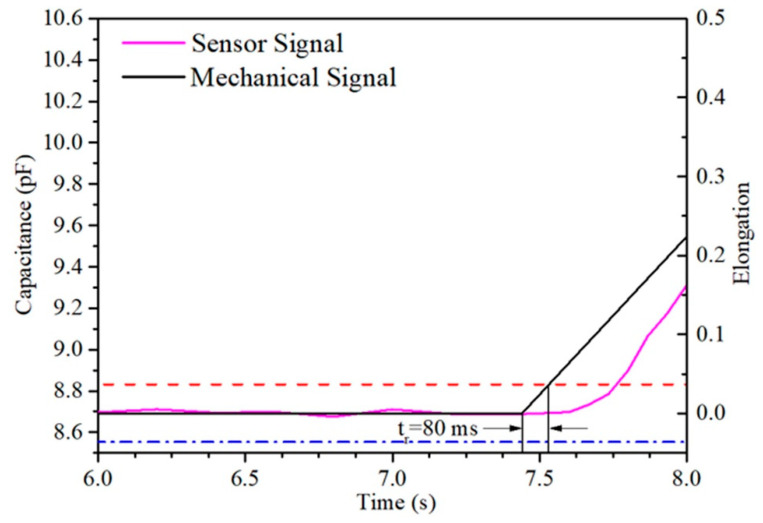
Sensor capacitance change over time during 0% to 40% elongation at 0.5 s^−1^ strain rate.

**Figure A9:**
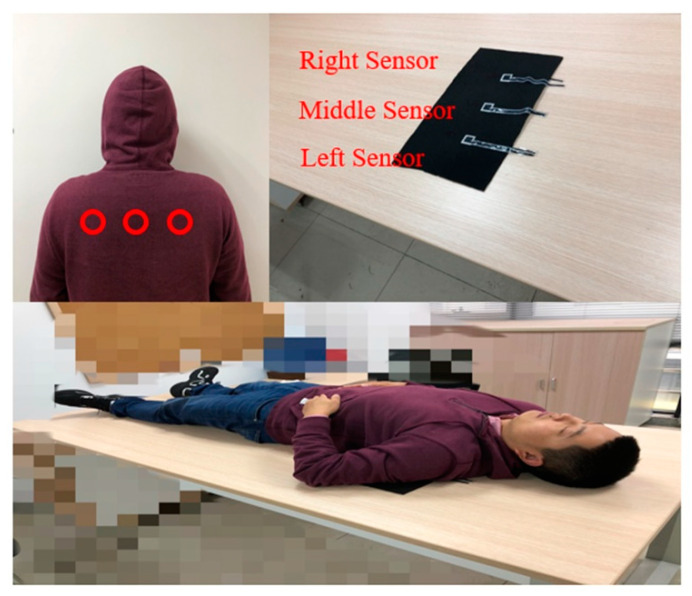
Sensor arrangement shows deep and normal breaths were recorded with the subject lying supine. The integrated sensor was placed under the back of the tester.

Clearly visible, during both walking and running, is the transition of force from the front of the foot to the heel, followed by the release as the heel is lifted. The delay between these motions is evident during walking, in Figure 9c, but much less so during running, in Figure 9d. Greater detail during these transitions may be gained in the future by acquiring the signal at a higher rate. Additionally apparent from running signals, is that the subject favors his left foot, with the pressure peaks typically higher for the left heel. Figure A6 gives photographic details of the sensor integration with an insole.

The combination of sensor durability and sensitivity when detecting foot motion, suggests a possible role in security applications such as intrusion detection—new ground for this class of sensor material.

**Figure 10:**
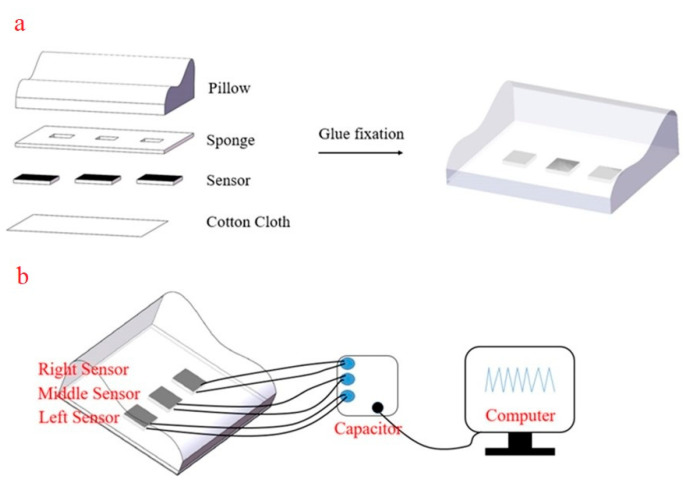
(**a**) Schematic of the three flexible sensors placed beneath the pillow, (**b**) test circuit diagram of the smart pillow.

**Figure 11:**
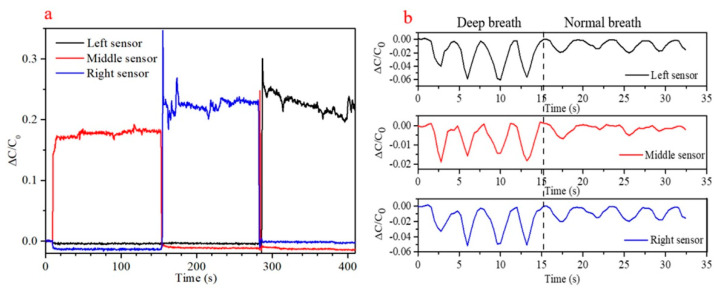
(**a**) Monitoring of head position with laterally separated sensors embedded under a pillow, as the head is moved from the center to the right hand side of the pillow, and then to the left. (**b**) Deep and normal breaths monitored with pad mounted sensors, laterally spaced.

Sensor integration with a test pillow is shown in Figure 10, where three sensors are positioned equidistantly across the lateral center line, at the bottom of the pillow. Changes in head position from pillow middle to right hand side and then to left hand side are clearly shown, with large changes in relative capacitance shown in Figure 11a. Such changes in head position have been used as simple indicators of sleep posture, including supine and lateral (log) positions [37].

Deep and normal breaths were recorded with the subject lying supine, with his head in the middle of the pillow, and back centered on the sensor pad. Individual breaths were clearly distinguished, with up to ~6% change in relative capacitance during the deep breathing phase. This corresponds with the results from Tian et al. [38], but with greater relative signal changes, in accordance with these sensors’ ~7× higher sensitivity. As may be expected given the concave lateral curvature of the back, the signal is stronger for the left and right sensors than for the center. Signal traces recorded during respiration with the three sensors are displayed in Figure 11b. This study has demonstrated that this class of relatively high sensitivity capacitive pressure sensors can fill a role previously held by piezoresistive sensors, and suggests the possibility of fulfilling an unmet medical need.

## 4. Conclusions

A porous silicone capacitive pressure sensor was developed, characterized, and applied to a wide variety of biosensing applications, including extension into the realm of physiological monitoring, via detection of head position and respiration sensing, while a subject lay on a mattress. In achieving this, the sensor was able to capitalize on its sensitivity to move into a class of applications typically occupied by resistive sensors, and in this case, an area of unmet medical need. The sensitivity displayed was in keeping with the high magnitude of its class, at ~0.1 kPa, with linearity of detection, low hysteresis, and robustness over 10,000 cycles of strain and compression also shown. Basic multiplexing and wearability were demonstrated with four channel devices, and potential for athletic monitoring was illustrated by integrating the suitably compliant material into the heel of an insole for pressure detection during walking and running—an impact mechanics situation. The robustness of the sensor, combined its sensitivity, and mechanical suitability for the latter role, suggests a possible future in security applications, as well as in biomedical fields.

## Figures and Tables

**Table 1:** Summary of performance of flexible capacitive strain and pressure sensors, reported in the literature, which do not possess a porous silicone dielectric material. Sensors were evaluated over five aspects: fabrication process, capacitive gauge factor (GF) for strain sensors, sensitivity over the 10 kPa range for pressure sensors, characterization as a wearable sensor, and characterization of sensor durability. Abbreviations: MWCNT—multi-walled carbon nanotube; CNT—carbon nanotube, CVD—chemical vapor deposition; SBS—poly(styrene-block-butadien-styrene); AgNP—silver nanoparticle; AgNW—silver nanowire.

Flexible Conductive Material	Ref.	Main Structure and Material	Sensor Type	G Factor	Sensitivity	Wearable Experiment	Durability Testing
CNT	[42]	MWCNT as conductive layer, Eraser as dielectric layer	Capacitive pressure	-	0.135 MPa^−1^	√	×
	[3]	CNT as conductive layer, PDMS layers fixed with silicone adhesive as dielectric layer	Capacitive Pressure and Strain	0.41	0.23 MPa^−1^	×	×
	[43]	CNT microyarn as conductive wire, Ecoflex as dielectric layer	Capacitive pressure	-	0.5 MPa^−1^	√	2000 cycles pressure
Graphene	[44]	graphene as conductive layer, PDMS as dielectric layer	Capacitive pressure	-	2 MPa^−1^	×	×
	[45]	CVD deposited graphene transferred to PDMS, nylon mesh dieletric and silver electrode sandwiched between two graphene-PDMS layers	Capacitive pressure	-	7 MPa^−1^	√	1050 cycles pressure
	[41]	Graphene oxide (GO) foam between reduced GO (rGO) patterned PET substrates	Capacitive pressure	-	150 MPa^−1^	×	1000 bending cycles
Carbon Black	[46]	Organo-silicone conductive silver adhesive serves as a flexible electrodes carbon black (CB)/silicone rubber (SR) composite dielectric	Capacitive pressure	-	0.2536 MPa^−1^	√	×
	[47]	Curing and gluing together successive blended PDMS/CB electrode layers and PDMS layers (dielectric, packaging)	Capacitive Strain	1	-	×	×
	This work	Carbon black conductive material PDMS sponge as dielectric layer	Capacitive Pressure and Strain	0.51	100 MPa^−1^ < 0.4 kPa, 49 MPa^−1^ > 0.4 kPa	√	10,000 cycles both strain and pressure
Nanowires	[4]	SBS/AgNP composite coated on the fiber as conductive fiber, PDMS as dielectric layer	Capacitive Pressure	-	64 MPa^−1^	√	10,000 bending cycles
	[48]	Transparent AgNW-PU composite as conductive layer, acrylic elastomer layer as the dielectric layer	Capacitive Pressure and Strain	0.5	3.3 MPa^−1^	×	10 cycles stretch
	[10]	AgNWs blend with PDMS as conductive layer, PDMS as dielectric layer	Capacitive Pressure and Strain	0.7	1.62 MPa^−1^	√	100 cycles stretch

**Table 2:** Summary of porous silicone dielectric based capacitive pressure sensors, and a notable polyurethane (PU) foam based sensor. Approximate sensitivity values are given for the 10 kPa range. Many of these sensors display much higher sensitivity in the range below ~2 kPa. Abbreviations: GNP—graphene nanoplatelets; SR—silicone rubber.

Ref.	Dielectric	Electrodes	Sensitivity (kPa^−1^)	Durability Cycles	Applications
[20]	Porous PDMS	AgNW + PDMS	0.057	50	Glove thumb tip pressure
[33]	PU foam + graphite	Silver coated nylon textile	0.05	-	Insoles
[49]	Plasma etched porous PDMS surface	AgNW + PDMS	0.57	3000	Glove finger tips, pressure mat array
[50]	GNPs/MWCNTs/SR coated PS sponge	silicone + Ag paste	0.033	2000	band: swallowing, breathing, pulse, muscle movement pad: air pressure, air flow, water drop or sand detection
[30]	Porous PDMS	ITO films	0.125	10,000	ant on band, pressure mat array
[29]	Porous PDMS	ITO/PET films	low range only	-	Pressure mat array
[12]	Porous PDMS coating of conductive fibers	Ag NP coated SBS coated Twaron fibers	0.104	10,000	Single sensor woven into mat
This Work	Porous PDMS	PDMS/CB blend	0.049 to 0.100	10,000	Pressure mat array, loading of band, insole, Pillow head position detection, and mattress insert for respiration

**Table 3:** Sensor strain sensitivity (GF) over 0% to 40% elongation, measured at different temperature and relative humidity (RH), and recorded at 0.5 Hz strain rate.

Test Conditions	Gauge Factor
20 °C, 50 RH%	0.51
20 °C, 60 RH%	0.52
20 °C, 70 RH%	0.53
25 °C, 60 RH%	0.51
30 °C, 60 RH%	0.50

**Figure A3:**
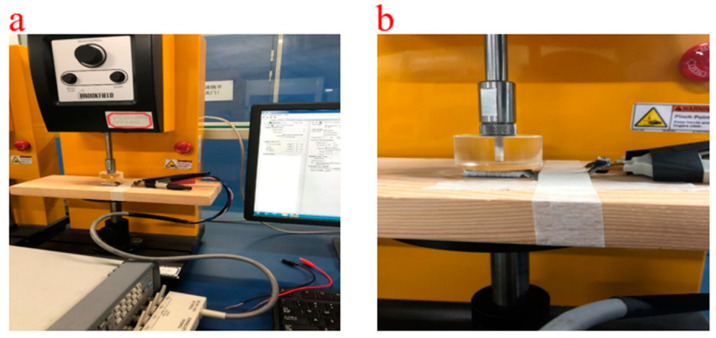
(**a**) Photo of the sensor before the compression test, (**b**) photo of the sensor under the compression test.

## Data Availability

Data available on request.

## Appendix A

Table A1 lists the different ratios of PDMS and CAM added. The volume reduction after ethanol soaking increases with the amount of CAM added. As the quantity of the CAM increases, the force required for the PDMS to be compressed by 50% decreases. It can be seen from the table, that when m_PDMS_:m_CAM_ = 1:9, the PDMS sponge displays a significant volume reduction. In the current work, we finally chose m_PDMS_:m_CAM_ = 1:7, in order to achieve a highly flexible sponge.

**Table A1:** Effect of the CAM addition on PDMS sponge.

Sample	m_PDMS_:m_CAM_	^a^ Volume Reduction/%	^b^ Force/N	Initial Volume/mm^3^	Volume after Ethanol Soaking/mm^3^
S1	3:1	22.3%	0.3	8800	6840
S2	5:1	22.3%	0.113	8800	6840
S3	7:1	47.5%	0.112	8800	4624
S4	9:1	56.5%	0.103	8800	3825

^a^: based on volume after ethanol soaking. ^b^: when the PDMS is compressed by 50%.

Figure A1 shows the the PDMS sponge with different mass fraction of CAM. From the images it is clearly to see in order to get high flexible PDMS sponge, we will choose the m_PDMS_:m_CAM_ = 1:7.

Figure A2b is the machine which was used in durability test. Figure A3c,d demonstrates the procedure.

Figure A3a is the photo of the sensor before the compression test, Figure A3b is the photo of the sensor under the compression test.

Figure A4 shows how the sensors were fixed on the arm, Figure A4a–d shows photos depicting when we pressed different number of the sensors. Figure A4e shows the sensors we made in this study.

Figure A5 shows how the sensors were fixed on the elbow, Figure A5a shows a photo of when the sensors were fixed on the elbow. Figure A5b shows the sensors we made in this study under stretching when the elbow was bending.

Figure A6 is a photo of placing of the sensor on the insole we made in this study.

Figure A7 shows the test applied of the flexible sensor on a pillow (making a smart pillow). Figure A7a shows three flexible sensors on the pillow, i.e., the left sensor, middle sensor and right sensor, respectively, Figure A7b is an image of our model system, which contains a collection chip and a computer.
